# Identification of Plant Virus Receptor Candidates in the Stylets of Their Aphid Vectors

**DOI:** 10.1128/JVI.00432-18

**Published:** 2018-06-29

**Authors:** Craig G. Webster, Elodie Pichon, Manuella van Munster, Baptiste Monsion, Maëlle Deshoux, Daniel Gargani, Federica Calevro, Jaime Jimenez, Aranzazu Moreno, Björn Krenz, Jeremy R. Thompson, Keith L. Perry, Alberto Fereres, Stéphane Blanc, Marilyne Uzest

**Affiliations:** a BGPI, Université de Montpellier, CIRAD, INRA, Montpellier SupAgro, Montpellier, France; b Université de Lyon, INSA-Lyon, INRA, BF2I, UMR0203, Villeurbanne, France; c Instituto de Ciencias Agrarias, Consejo Superior de Investigaciones Científicas, Madrid, Spain; d Plant Pathology and Plant-Microbe Biology Section, School of Integrative Plant Science, Cornell University, Ithaca, New York, USA; University of Maryland, College Park

**Keywords:** aphid, cuticular protein, plant virus, receptor, stylets, transmission

## Abstract

Plant viruses transmitted by insects cause tremendous losses in most important crops around the world. The identification of receptors of plant viruses within their insect vectors is a key challenge to understanding the mechanisms of transmission and offers an avenue for future alternative control strategies to limit viral spread. We here report the identification of two cuticular proteins within aphid mouthparts, and we provide experimental support for the role of one of them in the transmission of a noncirculative virus. These two proteins, named Stylin-01 and Stylin-02, belong to the RR-1 cuticular protein subfamily and are highly conserved among aphid species. Using an immunolabeling approach, they were localized in the maxillary stylets of the pea aphid Acyrthosiphon pisum and the green peach aphid Myzus persicae, in the acrostyle, an organ earlier shown to harbor receptors of a noncirculative virus. A peptide motif present at the C termini of both Stylin-01 and Stylin-02 is readily accessible all over the surface of the acrostyle. Competition for *in vitro* binding to the acrostyle was observed between an antibody targeting this peptide and the helper component protein P2 of Cauliflower mosaic virus. Furthermore, silencing the *stylin-01* but not *stylin-02* gene through RNA interference decreased the efficiency of Cauliflower mosaic virus transmission by Myzus persicae. These results identify the first cuticular proteins ever reported within arthropod mouthparts and distinguish Stylin-01 as the best candidate receptor for the aphid transmission of noncirculative plant viruses.

**IMPORTANCE** Most noncirculative plant viruses transmitted by insect vectors bind to their mouthparts. They are acquired and inoculated within seconds when insects hop from plant to plant. The receptors involved remain totally elusive due to a long-standing technical bottleneck in working with insect cuticle. Here we characterize the role of the two first cuticular proteins ever identified in arthropod mouthparts. A domain of these proteins is directly accessible at the surface of the cuticle of the acrostyle, an organ at the tip of aphid stylets. The acrostyle has been shown to bind a plant virus, and we consistently demonstrated that one of the identified proteins is involved in viral transmission. Our findings provide an approach to identify proteins in insect mouthparts and point at an unprecedented gene candidate for a plant virus receptor.

## Abstract (precis)

Most noncirculative plant viruses transmitted by insect vectors bind to their mouthparts. They are acquired and inoculated within seconds when insects hop from plant to plant. The receptors involved remain totally elusive due to a long-standing technical bottleneck in working with insect cuticle. Here we characterize the role of the two first cuticular proteins ever identified in arthropod mouthparts. A domain of these proteins is directly accessible at the surface of the cuticle of the acrostyle, an organ at the tip of aphid stylets. The acrostyle has been shown to bind a plant virus, and we consistently demonstrated that one of the identified proteins is involved in viral transmission. Our findings provide an approach to identify proteins in insect mouthparts and point at an unprecedented gene candidate for a plant virus receptor.

## INTRODUCTION

The transmission of plant viruses by aphid vectors has been classified into two major distinct categories designated “circulative” and “noncirculative” (1, 2). Circulative viruses are able to cross gut and salivary glands barriers. They are internalized in the aphid body, excreted into the salivary gland lumen, and finally inoculated via salivation into a new host plant. In contrast, noncirculative viruses do not penetrate the aphid body but are retained on the cuticle of the insect. They reversibly attach to the insect feeding apparatus during acquisition from an infected plant. There, they can remain infectious for only a short period (generally a few minutes) and are rapidly released and inoculated into a new host plant together with the aphid saliva (3–6). Most noncirculative virus species, including those in the families Bromoviridae, Potyviridae, Caulimoviridae, and Betaflexiviridae, are retained on the cuticle lining the inner canals of the maxillary stylets (7–10). However, a few species in the genera Sequivirus and Closterovirus are retained upstream in the foregut, either in the precibarium or in the cibarium (11, 12).

Numerous studies have attempted to decipher the molecular details of noncirculative virus-aphid interactions, but most have focused on viral determinants. These viral molecules are now well defined for the best-characterized viral species (for review, see references 5, 6, 13, and 14). Two types of proteins are key to successful interaction with aphid vectors: the coat protein and the virus-encoded “helper component.” Viruses in the genus Cucumovirus have adopted a “capsid strategy” and bind directly through their coat protein to putative receptors in aphid mouthparts (15–17), whereas a helper component is mandatory in the genera Potyvirus and Caulimovirus. This helper component—HC-Pro in the case of potyviruses and P2 in the case of Cauliflower mosaic virus (CaMV)—binds to putative receptors in insect stylets and creates a molecular bridge between the receptor and the virus particle (18–21).

To date, the identity of the receptors of noncirculative viruses in the aphid mouthparts (and/or foregut) is totally unknown. Their characterization is the major challenge to understanding noncirculative virus-vector interactions and to develop alternative control strategies that could interfere with virus transmission. The technical difficulty of manipulating insect stylets, a cell-free network of highly cross-linked chitin fibers and cuticular proteins, is undoubtedly the major bottleneck to solving this problem. Because noncirculative viruses are acquired when plant sap or cell contents are pumped through the stylets and released when saliva is spitted out, their receptors are usually assumed to be located at the extreme tip of aphid maxillary stylets, where the food and salivary canals fuse to form the common duct (8, 22). Consistently, using *in vitro* interaction assays, we earlier showed that the CaMV helper component P2 binds exclusively to cuticular proteins at the surface of the acrostyle, an organ discovered in the common duct of aphid stylets (9, 23). CPR proteins (cuticular proteins with the Rebers and Riddiford consensus sequence [RR]) represent the largest cuticular protein family described for arthropods (24, 25). In aphids, depending on the RR sequence, CPR proteins have been divided into subgroups RR-1, RR-2, and RR-3, RR-2 being by far the largest (25–30). Immunolabeling of dissected stylets revealed that proteins of the RR-2 subgroup are located within the acrostyle, but they proved poorly accessible at its surface and the identity of the corresponding genes could not be confirmed (23, 31).

To identify receptor candidates readily accessible at the surface of the acrostyle, we decided to extend this approach to the other subgroups of the CPR family. Here we report the identification of two closely related and highly conserved RR-1 cuticular proteins, Stylin-01 and Stylin-02, in the acrostyle of both Myzus persicae and Acyrthosiphon pisum. The two proteins harbor nearly identical C-terminal domains that are surface exposed and that perfectly matches the localization of the CaMV-P2 binding sites. Remarkably, CaMV P2 and an antibody targeting the surface-exposed domain of Stylin-01 and -02 competed for binding to the acrostyle, and RNA interference (RNAi) functionally confirmed that Stylin-01 is involved in CaMV transmission. Our results represent the first identification of cuticular proteins in aphid stylets and in the acrostyle and reveal Stylin-01 as the prime candidate receptor for the vector transmission of noncirculative plant viruses.

## RESULTS

### Identification of the first cuticular protein in aphid maxillary stylets: Stylin-01.

**FIG 1:**
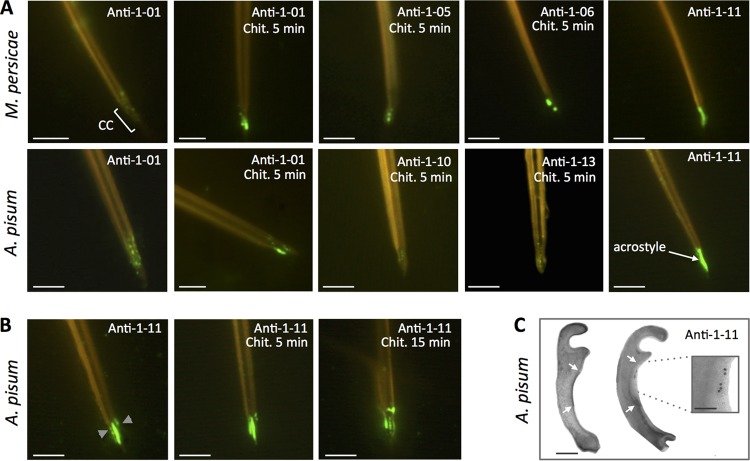
Stylin-01 is present in the acrostyle. (A) Immunolabeling of M. persicae (top) and A. pisum (bottom) dissected maxillary stylets untreated or predigested with chitinase (Chit.). Fluorescence signals were more or less intense depending on the antibody used and visible at the tip of maxillary stylets in the common canal (CC) and in the acrostyle. The antibodies for which no labeling has been observed are not shown (anti-08 and anti-09). (B) Anti-1-11 antibody directly labeled the surface of the acrostyle of aphid maxillary stylets and was weaker following a 5- to 15-min chitinase digestion treatment. (C) Transmission electron microscopy (TEM) observations of cross sections of the upper part of the common canal of A. pisum (indicated by gray arrowheads on the leftmost image of panel B). The darker density at the surface of the inner cuticle corresponds to the acrostyle (white arrows). Immunogold labeling with anti-1-11 antibody (right) showed gold particles at the surface of the acrostyle (magnified in the inset). Scale bars represent 5 μm in all immunofluorescence images, 100 nm in the TEM panel, and 50 nm in the inset.

We first produced the anti-1-01 antibody (Table 1), targeting the peptide 1-01 (ILVQDSAPSADGSLK), present in a single protein of M. persicae, MYZPE13164_G006_v1.0_000055990, and A. pisum, ACYPI009006. This protein is a typical RR-1 protein. When incubated with M. persicae or A. pisum individualized stylets, anti-1-01 antibody labeled the distal tip of maxillary stylets (Fig. 1A). The labeling was visible as fluorescent dots mainly in the acrostyle region, indicating that the corresponding protein is present at this location. However, the discontinuous and weak signal intensity suggested that the epitope recognized by anti-1-01 is embedded into the chitin and poorly accessible, as confirmed by the fact that a partial chitinase digestion prior to immunolabeling greatly increased the signal intensity (Fig. 1A).

**TABLE 1:** Antibodies and stylet immunolabeling results^*a*^

Antibody ID	Peptide ID	AA positions in proteins	Peptide sequence	Specificity	Labeling	Accessibility
Anti-1-01	1-01	32–46	ILVQDSAPSADGSLK	Stylin-01	+	PAc
Anti-1-05	1-05	21–35	PAGQSPESRAVILVQ	Stylin-01	+	Em
Anti-1-06	1-06	38–52	APSADGSLKNNFQTD	Stylin-01	+	PAc
Anti-1-07	1-07	32–46	SQEQEVNFDGNFKNK	Stylin-02	+	PAc
Anti-1-08	1-08	48–62	NFQTDNGIKQEEVRY	Stylin-01	−	NA
Anti-1-09	1-09	59–73	EVRYLKAGPEGPVSV	Stylin-01	−	NA
Anti-1-10	1-10	92–106	YVADENGYQPYGAHL	Stylin-01	+	Em
Anti-1-11	1-11	121–135	RYLASLPSTPEPKYQ	Stylin-01/02	+	Ac
Anti-1-13^*b*^	1-13	108–122	TPPPIPAEIQESLRY	Stylin-01/02	+	Em
		106–120	LPTPPPIPAEIQESL			

a ID, identifier; AA, amino acid; Ac, epitope exposed at the surface and directly accessible; PAc, epitope poorly accessible (labeling either weak or visible as dots without chitinase treatment); Em, epitope embedded, not accessible at the surface of the stylets (labeling visible only after chitinase digestion); NA, not applicable, because of the total lack of labeling regardless of the treatment. +, labeling observed at the tip of maxillary stylets; −, no labeling detected regardless of the tested conditions.

b Due to poor immunogenicity prediction, two partially overlapping peptides were used for immunization.

To further confirm the presence of this protein at the tip of M. persicae and A. pisum maxillary stylets, and to evaluate whether one or more of its epitopes may emerge at the surface and be readily accessible, seven additional antibodies were produced (Table 1). The targeted peptides were designed in order to completely cover the mature protein (116 amino acids in length). Unfortunately, an 18-amino-acid region within the RR-1 chitin-binding domain was predicted to be poorly immunogenic and thus not considered for peptide syntheses and antibody production. Antibodies anti-1-08 and anti-1-09 did not label M. persicae or A. pisum stylets, even after partial chitinase treatment. All other antibodies (anti-1-05, anti-1-06, anti-1-10, anti-1-13, and anti-1-11) positively labeled the tip of M. persicae and/or A. pisum maxillary stylets (Fig. 1A), with a remarkable intensity for anti-1-11. The corresponding protein in each aphid species was, respectively, named Mp_Stylin-01 and Ap_Stylin-01.

### Peptide 1-11 is directly accessible at the surface of the acrostyle.

In contrast to all other peptides of Stylin-01 (Fig. 1A), the peptide 1-11 (RYLASLPSTPEPKYQ) is readily accessible without the need for chitinase treatment (Fig. 1B) and completely covers the surface of the acrostyle. Interestingly, in this case, the intensity of the labeling was weakened by chitinase digestion. The presence of peptide 1-11 was also revealed on the sides of the food and common canals at the tip of maxillary stylets, indicating that it may emerge not solely from the acrostyle but also from neighboring regions (Fig. 1B). In parallel, gold labeling of ultrathin cross sections of A. pisum maxillary stylets with anti-1-11 further confirmed the presence of the corresponding peptide at the surface of the acrostyle (Fig. 1C).

### Stylin-01 is highly similar to another RR-1 protein.

**FIG 2:**
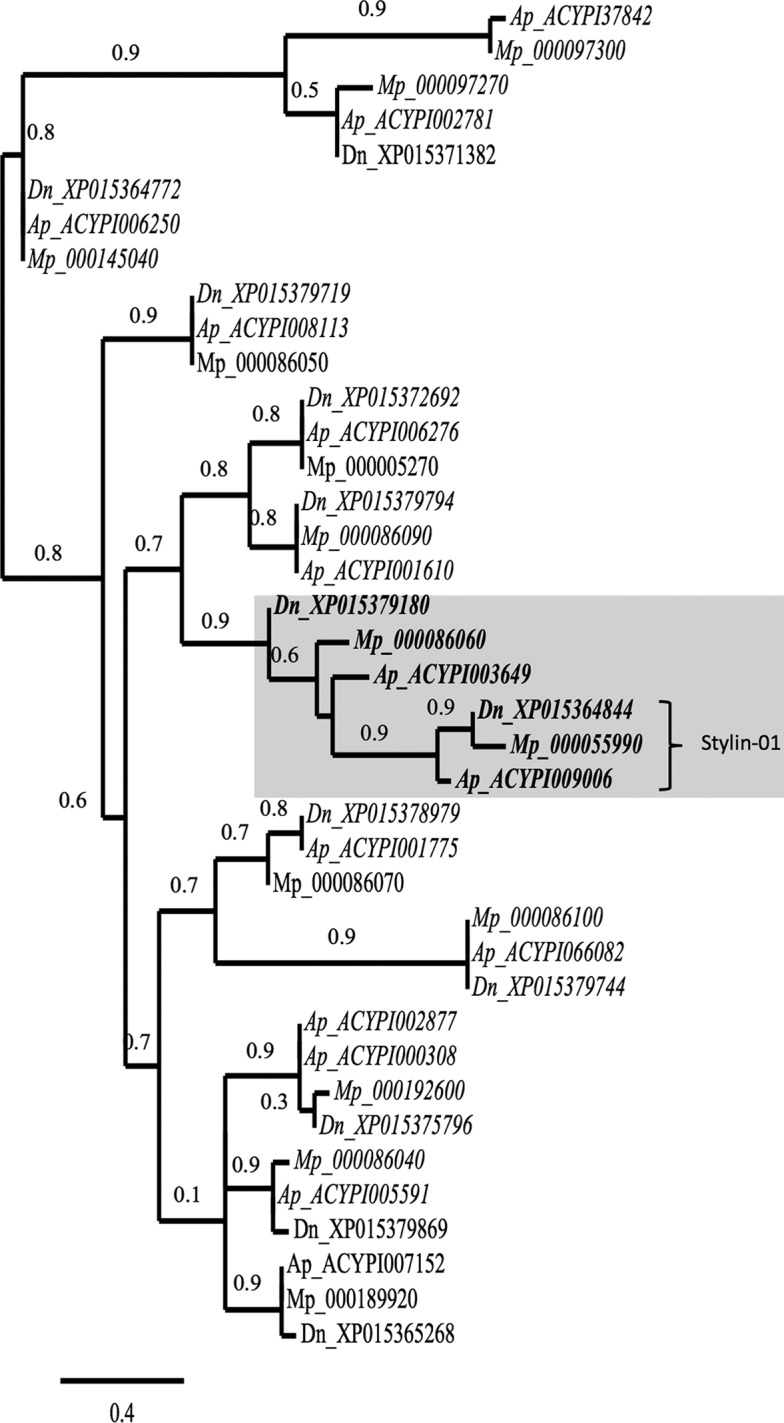
Phylogenetic relationships of putative RR-1 proteins of M. persicae (Mp), A. pisum (Ap), and D. noxia (Dn) with trimmed signal peptides. Database accession numbers for each gene are indicated on the right of the species abbreviation. It is noteworthy that to retrieve M. persicae sequences from Aphidbase, numbers should be preceded by MYZPE13164_G006_v1.0_. Gene orthologs determined by OrthoMCL software are in italics. Branch support values are indicated at the node, and the scale bar represents probabilities of change from one amino acid to another in terms of a unit, which is an expected 1% change between two amino acid sequences. Stylin-01 proteins form a cluster shaded with Ap_ACYPI003649, Mp_000086060, and Dn_XP015379180.

**FIG 3:**
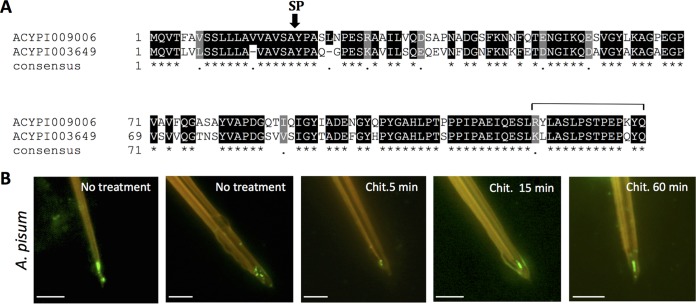
ACYPI003649, a second RR-1 protein identified in the acrostyle. (A) Alignments of Ap_ACYPI009006 (Ap_Stylin-01) and Ap_ACYPI003649 were performed using T-Coffee software (66, 67). Shading was done with BOXSHADE 3.21 software. Identical residues are shaded in black; similar residues are shaded in gray. The consensus sequence is shown at the bottom, with periods indicating conserved substitutions and asterisks indicating identities. The signal peptide (SP) cleavage site is indicated. The line above the sequence delineates peptide 1-11. (B) Immunolabeling of A. pisum stylets with anti-1-07 antibody. Scale bars represent 5 μm.

A neighbor-joining phylogeny analysis revealed orthologs for almost all RR-1 cuticular proteins in the three aphid species with available genome sequences, M. persicae (Mp), A. pisum (Ap), and Diuraphis noxia (Dn). Furthermore, the phylogenetic tree illustrated that Stylin-01 clearly grouped with a second RR-1 protein: Ap_ACYPI003649*/*Mp_G006_000086060/Dn_XP_015379180.1 (Fig. 2). In this analysis, the mature protein sequences of Ap_ACYPI009006 (Ap_Stylin-01) and Ap_ACYPI003649 were 71% identical. Moreover, the 15 C-terminal amino acids of this closely related RR-1 protein shared 80% identity (12 out of 15 amino acid residues) with peptide 1-11 of Stylin-01 (Fig. 3A). This high degree of identity between the C-terminal sequences of Stylin-01 and the second protein prompted testing whether anti-1-11 could recognize both and therefore whether Ap_ACYPI003649 could also be present in aphid stylets. Peptides of 18 amino acids in length containing the 15 C-terminal amino acids of Ap_ACYPI009006 (Stylin-01 peptide 1-11) and Ap_ACYPI003649 were blotted onto nitrocellulose membrane for Western blot analyses. In contrast with anti-1-01 antibody, which reacts specifically with the N terminus of Ap_ACYPI009006 and not with that of Ap_ACYPI003649, this experiment revealed that anti-1-11 antibody reacts strongly with both peptides (Table 2). Therefore, the acrostyle labeling observed with anti-1-11 could be due to the C terminus of Ap_ACYPI009006 (Stylin-01), of Ap_ACYPI003649, or both.

**TABLE 2:**
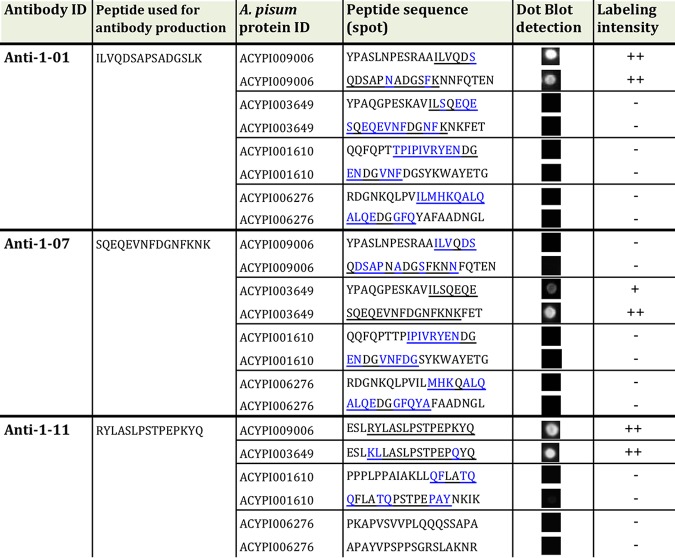
Specificities of anti-1-01, anti-1-11, and anti-1-07 antibodies assessed by dot blot analyses on peptides originating from proteins closely related to ACYPI009006 and ACYPI003649^*a*^

a −, no labeling; +, weak labeling; ++, strong labeling.

### Identification of a second RR-1 cuticular protein in aphid maxillary stylets – Stylin-02.

While Ap_ACYPI003649 is recognized by the anti-1-11 antibody, this does not necessarily confirm that this protein is also present in aphid stylets. To assess this possibility, an antibody specifically directed to Ap_ACYPI003649 protein was produced. A peptide of 15 amino acids in length—peptide 1-07 (Table 1)—was chosen in the N-terminal part of the mature protein for immunization, in the region where sequences of Ap_ACYPI003649 and Ap_ACYPI009006 are the most divergent. A dot blot analysis confirmed the specificity of anti-1-07 antibody for the corresponding peptide of Ap_ACYPI003649 (Table 2). When incubated with A. pisum dissected stylets, anti-1-07 antibody labeled the tip the acrostyle (Fig. 3B), indicating the presence of the Ap_ACYPI003649 protein that we logically named Stylin-02. As for regions of Stylin-01 other than the C terminus, the labeling of Stylin-02 with anti-1-07 seemed stronger after an extended treatment (15 to 60 min) with chitinase, indicating that this motif is poorly accessible at the surface (Fig. 3B).

Based on these results, it is impossible to conclude about the relative contribution of Stylin-01 or Stylin-02 to the strong labeling at the surface of the acrostyle observed with anti-1-11.

### Stylin-01 and Stylin-02 are conserved among aphid species.

A comparative analysis was conducted on Stylin-01 and Stylin-02 homologs found in several aphid species (see Fig. S1A and B in the supplemental material), through BLAST searches or direct sequencing of aphid colonies maintained in our laboratory (accession numbers are given in Table S1 in the supplemental material). For both proteins, high sequence similarities were found across aphid species, with the RR-1 chitin-binding domain being almost identical. Remarkably, the amino acid sequences of the C-terminal domains were nearly identical in Stylin-01 and Stylin-02 (containing peptide 1-11) in all aphid species investigated.

### CaMV-P2 and anti-1-11 compete for binding to the acrostyle.

**FIG 4:**
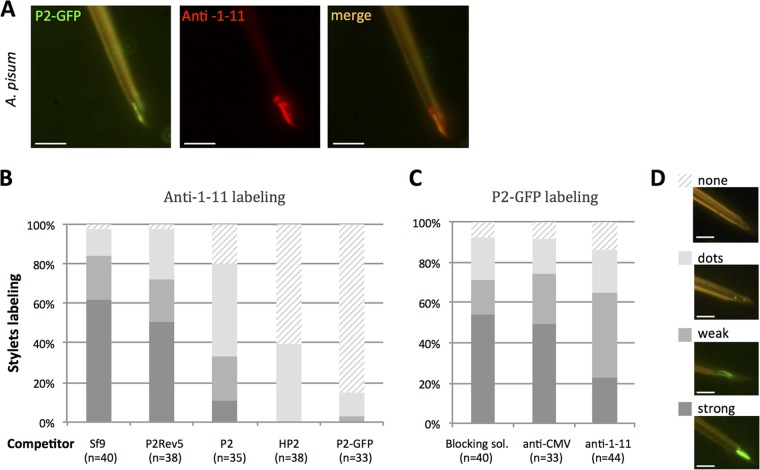
CaMV protein P2 and anti-1-11 IgGs colocalize in and compete for the acrostyle. (A) Coincubation of P2-GFP and anti-1-11 antibody with A. pisum dissected stylets. P2-GFP (green fluorescence) and anti-1-11 antibody (red fluorescence) colocalize on the acrostyle (seen as orange labeling). (B) Histograms show the proportion of maxillary stylets with the acrostyle labeled by anti-1-11 antibody from 4 independent experiments, after preincubation with crude extracts from healthy Sf9 cells (Sf9), from Sf9 cells producing P2, P2Rev5 mutant, P2-GFP fusions, or purified His tag-P2 (HP2), as indicated. While no significant difference was observed in the intensity of anti-1-11 labeling after Sf9 or P2rev5 preincubation (one-way ANOVA, *P* = 0.779), the proportion of stylets strongly labeled was significantly reduced when P2, P2-GFP, and HP2 were used as competitors (one-way ANOVA, *P* = 2.27 × 10^−5^). (C) Histograms show the proportion of maxillary stylets with the acrostyle labeled by P2-GFP from 4 independent experiments, after preincubation with blocking solution containing no antibody, anti-CMV as a negative control, and anti-1-11 antibodies. The intensity of the labeling observed after preincubation with blocking solution containing anti-1-11 antibodies is reduced compared to that with stylets preincubated with blocking solution or anti-CMV as a negative control. However, this reduction is not significant (ANOVA, *P* = 0.0503). The numbers of maxillary stylets observed for each treatment is indicated as “n.” Each labeled stylet counted in panels B and C was scored as strongly, weakly, dot, or not labeled; a representative image illustrating each of these scores is shown in panel D. Scale bars represent 5 μm.

The coincubation of dissected stylets of A. pisum with P2 fused to green fluorescent protein (P2-GFP) and anti-1-11 antibody (detected with Alexa-594 conjugated secondary antibodies) indicated a near-perfect colocalization of their respective target (Fig. 4A).

When stylets of A. pisum were preincubated with the viral protein P2 or derivative fusions (P2-GFP and an N-terminal 6-histidine tag fusion, HP2), the subsequent binding of anti-1-11 antibody onto the acrostyle was significantly reduced or abolished compared to nonbinding pretreatment controls (Sf9 cell crude extracts or the acrostyle-nonbinding P2Rev5 mutant [9]) (Fig. 4B). Consistently, but to a lesser extent, this occurred in a reversed-competition experiment, when stylets of A. pisum were preincubated with anti-1-11 the subsequent binding of P2-GFP fusion was similarly hindered, whereas nonbinding pretreatment controls (blocking solution or anti-CMV, an antibody unrelated to aphid proteins) had no effect (Fig. 4C).

Taken together, these results demonstrate that the viral protein P2 and the specific anti-1-11 antibody compete *in vitro* for binding to the acrostyle.

### Silencing of Mp_Stylin-01 gene expression decreases CaMV transmission efficiency.

**FIG 5:**
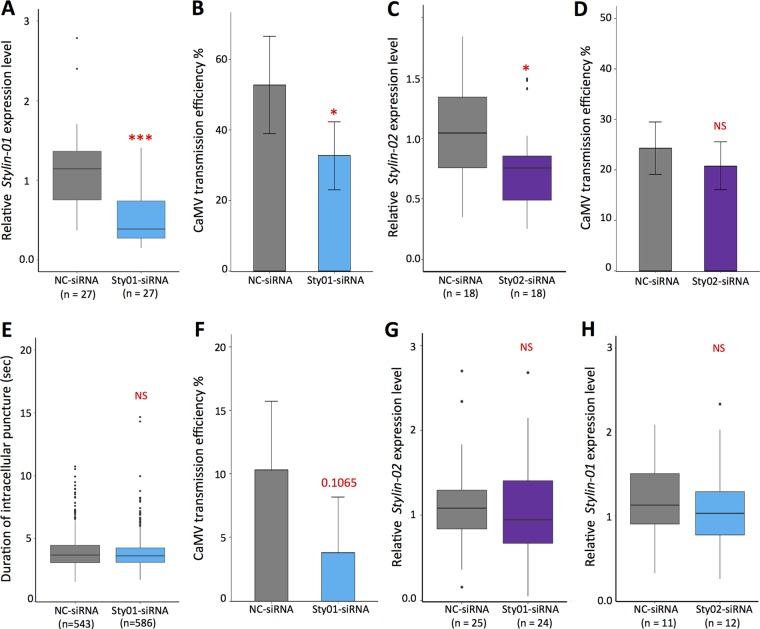
Effects of *stylin-01* and *stylin-02* gene silencing on cauliflower mosaic virus transmission. (A) Box plots represent median (horizontal line) and 25th and 75th percentiles of transcript levels of *stylin-01* normalized against two reference genes, the actin and EF1α genes. The *stylin-01* relative expression level is significantly reduced in *sty01*-siRNA-treated Myzus persicae compared to the NC-siRNA group (ANOVA, *n* = 27 pools of 10 aphids each, *P* = 8.12 × 10^−6^). (B) Proportion of CaMV-infected plants calculated from two independent experiments (means ± SDs). For each experiment and for each treatment, two aphids were transferred onto each test plant (*n* = 48) after a 5-min acquisition access period (AAP). The asterisk indicates significant differences between virus transmission efficiencies (chi-square test, *P* = 0.016). (C) Box plots represent *stylin-02* transcript levels normalized against two reference genes, as in panel A. The *stylin-02* relative expression level is significantly reduced (ANOVA, *n* = 18 pools of 10 aphids, *P* = 0.0231). (D) Proportion of CaMV-infected plants calculated from three independent experiments (means ± SDs). For each experiment and for each treatment, as for panel B, we used two aphids per test plant (*n* = 72). No significant difference was observed between virus transmission efficiencies (chi-square test, *P* = 0.473). (E) Boxes showing the interquartile and the median represent the mean duration of intracellular punctures (pds) produced on CaMV-infected turnip plants by M. persicae fed for 72 h on NC-siRNA (*n* = 543) or *sty01*-siRNA (*n* = 582). No significant difference was observed between the two treatments (ANOVA, *P* = 0.574). (F) Proportion of CaMV-infected plants calculated from five independent experiments (means ± SDs). For each experiment repeat and each treatment, a single aphid was transferred per test plant (*n* = 22) after an AAP of 5 pds. The *P* value (chi-square test) reflecting a marginally significant difference between the two treatments is indicated on the graph. Black circles in panels A, C, and E are outliers. (G and H) Controls demonstrate that Sty01-siRNA and Sty02-siRNA do not silence stylin-02 (G) and stylin-01 (H), respectively. The specificity of silencing of Sty01-siRNA was estimated from 25 and 24 pools (for NC-siRNA and Sty01-siRNA, respectively) out of the 27 pools used for panel A and from 11 and 12 pools (for NC-siRNA and Sty02-siRNA, respectively) out of the 18 pools used for panel C. Asterisks in panels A and C indicate differences as follows: ***, *P* < 0.001, and *, *P* < 0.05. NS, not significant.

The impact of Stylin-01 and Stylin-02 on CaMV transmission efficiency was investigated using RNAi-mediated silencing on the vector species M. persicae. When cohorts of first-instar nymphs were fed with small interfering RNAs targeting specifically *stylin-01* transcripts (*Sty01*-siRNA), their accumulation was significantly reduced (by 66.1%) compared to that in cohorts of nymphs fed with a negative-control siRNA (NC-siRNA; Fig. 5A). Interestingly, *Sty01*-siRNA-treated aphids transmitted CaMV at a significantly lower rate (reduced by 40%) than did aphids fed with NC-siRNA (Fig. 5B).

When aphids were fed on artificial diets containing siRNA that specifically target *stylin-02* transcripts (*Sty02*-siRNA), their accumulation was also significantly reduced (by 27.6%) compared to that in cohorts of nymphs fed with NC-siRNA (Fig. 5C). However, in that case, the decrease in *stylin-02* mRNA did not lead to a significant change in CaMV transmission efficiency (Fig. 5D).

Therefore, even though both *stylin-01* and *stylin-02* transcript levels were significantly reduced, only Stylin-01-silenced aphids showed a significant decrease in their ability to transmit CaMV.

While these results suggest that reducing the *stylin-01* mRNA level may have a direct impact on CaMV transmission by decreasing the amount of Stylin-01 protein in aphid stylets, the above-described experiments do not exclude a possible indirect effect of silencing on aphid feeding behavior and therefore on virus acquisition. The probing behavior of aphids was monitored to assess a potential effect of Stylin-01 silencing on the duration of intracellular punctures (potential drop waveforms [pds]) on CaMV-infected turnips. It is known that intracellular punctures are mandatory for the acquisition of CaMV and that their duration is positively correlated with the efficacy of ulterior transmission (32). Remarkably, over more than 500 recorded potential drops for silenced and for nonsilenced aphid cohorts, the mean durations of intracellular punctures were nearly identical, with respective averages (means ± standard errors of the means [SEMs]) of 3.96 ± 1.32 s and 3.91 ± 1.76 s (Fig. 5E), indicating that Stylin-01 mRNA depletion does not impact on this trait closely linked to virus transmission.

During these experiments, after successfully completing 5 pds, each aphid was individually transferred to healthy turnip plants to check their virus transmission ability. It is noteworthy that CaMV transmission rates were low for all biological replicates under these experimental conditions, reducing the statistical power of our test. Nevertheless, a consistent trend of 50 to 100% reduction of CaMV transmission efficiency was observed in the experimental repeats for Stylin-01-silenced aphids (Fig. 5F).

A logical way to explain that silencing of Stylin-01 decreases CaMV transmission is that it decreases the binding of P2 onto the acrostyle. For practical reasons, such semiquantitative estimates could only be carried out on the larger stylets of A. pisum. Silenced A. pisum (see Materials and Methods) were scored as in Fig. 4D for intensity of P2-GFP binding. The results obtained from two independent experiments show a strong reduction of P2-GFP binding (Table 3).

**TABLE 3:** P2-GFP labeling of dissected stylets of Acyrthosiphon pisum silenced for *stylin-01* gene expression

RNAi treatment	P2-GFP labeling^*a*^				
	Strong	Weak	Dots	None	Total
NC-siRNA	19 (79.2)	4 (16.6)	0 (<4.2)	1 (4.2)	24 (100)
Sty01-siRNA	1 (6.7)	0 (<6.7)	0 (<6.7)	14 (93.3)	15 (100)

a The percentage of maxillary stylets labeled out of the total number of maxillary stylets observed is indicated in parentheses.

## DISCUSSION

The identification of virus receptors within their insect vectors is a major challenge faced by virologists studying viruses in both the animal and plant kingdoms. Several candidate proteins have earlier been shown to interact with plant viruses using various approaches, such as far-Western blot (33–38), yeast-two hybrid (39, 40), or genomics, transcriptomics, and proteomics (39, 41–45) approaches. However, their actual role as receptors has proven difficult to validate. To date, the most comprehensive and convincing study concerns Pea enation mosaic virus (PEMV; Luteoviridae), transmitted by A. pisum in a circulative manner. The coat protein of PEMV directly interacts with aminopeptidase N (APN) of the aphid gut cell membrane (46, 47). A pea aphid gut binding peptide, GBP3.1, competes with PEMV for binding to APN and reduces viral accumulation in the aphid hemocoel (46, 48). The authors therefore concluded that APN functions as a gut receptor for PEMV in the pea aphid (47). For stylet-borne noncirculative viruses, the putative receptors are cuticular proteins entangled with the chitin fibers of the stylet (23), a cell-free structure (49), and they are notoriously difficult to extract. Such a technical hurdle has long obstructed the elucidation of the molecular dialogue between noncirculative viruses and their insect vectors.

Here we identify two proteins in the aphid stylets, and more generally in arthropod mouthparts, Stylin-01 and Stylin-02. These proteins share more than 70% identity and have nearly identical C-terminal domains. They are conserved at least in the subfamilies Aphidinae, Lachninae, and Eriosomatinae, the Aphidinae subfamily being the one in which almost all vectors of noncirculative viruses have been described (50). While anti-1-08 and anti-1-09 did not label aphid stylets under our experimental conditions, probably due to a lack of accessibility of the corresponding peptides within the superficial layers of the stylet cuticle, we extended the list of the peptides successfully detected within the acrostyle (23, 31). Although we could not distinguish whether Stylin-01, Stylin-02, or both actually display their conserved C termini at the surface of the cuticle, our immunolabeling approach definitely demonstrated that the C-terminal protein domain revealed by anti-1-11 covers all the surface of the acrostyle. It is thus in contact with ingested plant sap and secreted aphid saliva and has the potential to play a key role in virus retention and release.

RNAi-mediated interference in aphids has been attempted in several studies (51, 52). The efficacy of silencing is notoriously highly variable and dependent on the gene targeted (52, 53). Unfortunately, a complete knockdown could never be achieved whatever the method and aphid species used and the gene targeted (51). In our study, oral delivery of specific siRNA significantly reduced the expression of the *stylin-01* and, to a lesser extent, *stylin-02* genes. Reducing the expression of Stylin-01 impacted the ability of aphid vectors to transmit CaMV but did not alter the aphid feeding behavior during the probing events associated with virus uptake. Therefore, it is tempting to conclude that Stylin-01 may act as a receptor and bind directly the CaMV helper protein P2. Consistent with this proposition is the demonstration that P2 and the antibody directed against the surface-exposed C terminus of Stylin-01 compete for attachment to the acrostyle. However, other possibilities cannot totally be excluded at this stage. For example, reducing the amount of Stylin-01 protein within aphid stylets could also locally perturb the ultrastructure of the acrostyle, affecting the accessibility of active domains of receptor molecules other than Stylin-01. To definitely confirm that Stylin-01 is the receptor driving CaMV transmission, direct *in vitro* binding to CaMV-P2 would be necessary. Unfortunately, Stylin-01 and Stylin-02 proteins were totally unfolded under the numerous experimental *in vitro* conditions we assessed. It is possible that cuticular proteins fold solely in the presence of chitin-chitosan fibers, in which case *in vitro* binding between Stylin proteins and CaMV-P2 would be extremely difficult to realize, if not impossible.

Our results indicate that in contrast to Stylin-01, Stylin-02 is unlikely to be involved in CaMV transmission, although it may be argued that *stylin-02* knockdown was not effective enough to observe a phenotype. We can speculate that Stylin-02 is less abundant within the acrostyle. Reducing its amount in aphid stylets may, then, affect only partially the function of the acrostyle, and we could not detect any defect of the silenced aphid in CaMV transmission. Another hypothesis relies on the few differences in amino acid composition between the Stylin-01 and Stylin-02 C-terminal domains. Investigating whether these subtle differences could explain the lack of effect of Stylin-02 on CaMV transmission will require a technical means (not yet defined) to distinguish between the C-terminal domains of these proteins.

Recently, Liang and Gao (54) reported a positive interaction in the yeast-two-hybrid system between the coat protein of a noncirculative virus, Cucumber mosaic virus (CMV; isolate SXCH [GenBank JX993913]), and Mpcp4 of their M. persicae colony from southeastern China. Mpcp4 was first identified in 2007 by Dombrovsky et al. (37) during a search for extractible cuticular proteins in whole aphids. This protein actually corresponds to the protein we identified in this study as Stylin-01. Liang and Gao (54) concluded that “Mpcp4 gene promoted CMV acquisition in M. persicae,” but a formal demonstration of its role in CMV transmission was not provided to support this conclusion. We here show that Stylin-01 solely emerges at the tip of the maxillary stylets, in the acrostyle, and the binding site of CMV to the aphid's cuticle has not been precisely localized. We have thus far failed to attach this virus to the acrostyle, despite a large range of conditions tested, and so whether Stylin-01 is involved in the transmission of CMV remains an open question. Likewise, and more generally, whether Stylin-01 and possibly also Stylin-02 are receptors of noncirculative viruses other than CaMV will need further investigations.

In this report, we provide new insight into the proteomic composition of the acrostyle and, for the first time, key information on its surface composition, which may lead to a better understanding of its functions. In addition to the previously reported RR-2 protein(s) (23, 31), we show that the acrostyle contains two RR-1 proteins, Stylin-01 and Stylin-02. Our *in vitro* competition and *in vivo* silencing approaches strongly support the role of Stylin-01 as the receptor of CaMV. Moreover, beyond the case of the transmission of CaMV by aphid vectors, we here illustrate the potential complexity of the acrostyle at the virus-vector and plant-insect interface and provide an efficient method to further search and characterize all proteins emerging at the surface of arthropod mouthparts. It must be noted that these proteins are of interest for the transmission of all noncirculative viruses that sum up to hundreds of species of major economic importance.

## MATERIALS AND METHODS

### Aphid clones.

All aphid colonies were maintained in growth chamber at 23/18°C (day/night), with a photoperiod of 16/8 h (day/night). Acyrthosiphon pisum LL01 and Myzus persicae Sulzer were maintained on Vicia faba cv. Aguadulce, and on Solanum melongena cv. Barbentane, respectively.

### Antibodies.

Antibodies used in the present study were produced against peptides originating from cuticular proteins of the CPR family of M. persicae and/or A. pisum (Table 1), proteins from the CPR family having high degrees of identity between orthologs in these two aphid species (30). Peptides synthesized by Eurogentec (Kanaka Eurogentec S.A., Seraing, Belgium) were used to immunize rabbits. Antisera were collected and purified on the peptides used for immunization. Primary antisera were used at dilutions of 1:200 for immunolabeling of stylets and 1:1,000 for Western blot analyses. Secondary antibodies Alexa Fluor 488- and Alexa Fluor 594-conjugated anti-rabbit IgG (A11070 and A11012; Thermo Fisher Scientific, Waltham, MA) were used at a dilution of 1:800, goat anti-rabbit (GAR) IgG-horseradish peroxidase (HRP) (sc-2030; Santa Cruz Biotechnology, Dallas, TX) was used at a dilution of 1:5,000, and 10-nm colloidal gold-conjugated GAR IgG (BBI Solutions, Cardiff, UK) was used at a dilution of 1:25. Polyclonal Cucumber mosaic virus (CMV) antibodies (CAB 44501/0500; Agdia Inc., Elkhart, IN) were used at a dilution of 1:200 in competition experiments.

### Protein production.

The P2 helper protein of CaMV (isolate Cabb B-JI), either native or fused to green fluorescent protein at its C terminus (P2-GFP), and the nontransmissible P2Rev5 derivative mutant (55) were expressed in the Sf9 baculovirus/insect cell system as previously described (9). Insect cells harvested 48 h after infection with a baculovirus recombinant were resuspended in DB5 buffer {50 mM HEPES (pH 7.0), 500 mM LiSO_4_, 0.5 mM EGTA, 0.2% (wt/vol) 3-[(3-cholamidopropyl)-dimethylammonio]-1-propanesulfonate (CHAPS)}. Cells were lysed by freeze-thaw cycling at −20°C. Cellular debris was removed by centrifugation. Alternatively, P2 fused to a hexahistidine N-terminal tag (HP2) was also produced and was affinity purified on nickel-nitrilotriacetic acid (Ni-NTA) resin as described previously (56). Aliquots of 50 μl of crude extracts or purified proteins were kept at −20°C until use.

### Peptide dot blot analyses.

Peptides of 18 amino acids, comprising either the N termini (mature proteins) or the C termini of Ap_ACYPI009006, Ap_ACYPI003649, Ap_ACYPI001610, and Ap_ACYPI006276, were synthesized by Proteomic Solutions (Saint-Marcel, France). Peptides were detected by Western blot analysis using anti-1-01, anti-1-07, or anti-1-11 antibodies.

### *In vitro* interactions on dissected individualized stylets.

Individualized M. persicae and A. pisum stylets were immunolabeled according to the methods of Uzest et al. (23) and Webster et al. (31). Dissected stylets preincubated in blocking solution (TBS buffer [50 mM Tris and 200 mM NaCl, pH 7.4] supplemented with 1% [wt/vol] skim milk powder and 0.05% [vol/vol] Tween 20) for 20 min were incubated in primary antibodies for 14 h, followed by 4 h of incubation in secondary Alexa Fluor-conjugated antibody at room temperature. In addition, stylets were pretreated with 2 U/ml of chitinase from Streptococcus griseus (Sigma) in 50 mM phosphate buffer (pH 6.0) at room temperature. Alternatively, stylets were incubated with 5 μl of P2-GFP crude extracts along with the primary anti-1-11 antibody for colocalization experiments, followed by secondary antibody incubations as described above.

A series of sequential incubations onto dissected stylets was done using the baculovirus/Sf9 cell-produced viral proteins P2, P2Rev5, HP2, and P2-GFP as primary interacting molecules. A crude extract of healthy Sf9 cells was included as a negative control. Twenty microliters of these primary interacting molecules was incubated for 14 h in modified DB5 buffer (DB5-0.05 containing 0.05% [wt/vol] CHAPS [final concentration]). Slides were rinsed once in DB5-0.05 buffer and twice in TBS buffer. Stylets were incubated in anti-1-11 primary antibody as described above for 1 h. The secondary antibody used to reveal the presence of anti-1-11 on the stylets was Alexa Fluor 594-conjugated antibody when P2-GFP was used as the primary interacting molecule; Alexa Fluor 488-conjugated antibody was used for all other experiments. The incubation time was also reduced to 1 h. A second series of sequential incubations onto dissected stylets was performed using anti-1-11 antibody as the primary interacting molecule. In this case, the blocking solution alone, or the blocking solution containing a serum unrelated to aphid proteins (anti-CMV), was used as a negative control. Incubation was followed by one rinse with TBS buffer and two rinses with DB5-0.05 buffer. Stylets were finally incubated with 5 μl of P2-GFP crude extracts in DB5-0.05 for 2 h. Four independent replicates were performed for each experiment and for all treatments. Observations were done with an Olympus BX60 microscope equipped for epifluorescence.

### Sample preparation for electron microscopy and immunogold labeling.

The anterior parts of anesthetized A. pisum heads were severed in fixation buffer (FB; 0.1 M cacodylate buffer [pH 7.2]) under a dissecting microscope using razor blades. Samples were fixed in FB containing 0.5% glutaraldehyde and 2% paraformaldehyde and embedded in LR Gold resin. Primary antisera and secondary antibodies were used at a 1:25 dilution. Sections (60 nm thick) were observed in a JEOL 100CXII microscope operated at 60 to 80 kV.

### RNA interference.

As stylets are deprived of cells, RNAi treatments were applied at earlier larval stages and expected phenotypes were evaluated after stylet *de novo* synthesis, i.e., after at least one molting event from the start of short interfering RNA (siRNA) delivery. RNAi treatments were carried out on 3-day-old synchronized M. persicae aphids. Since *stylin-01* and *stylin-02* genes share 74% identity, we paid particular attention while designing short interfering RNAs to specifically target one or the other gene (Kanaka Eurogentec S.A.). Negative control siRNA (NC-siRNA; SR-CL000-005, Eurogentec), siRNA targeting specifically *stylin-01* mRNA (*Sty01*-siRNA; sequences are described in Table S2 in the supplemental material), or siRNA targeting specifically *stylin-02* mRNA (*Sty02*-siRNAa and *Sty02*-siRNAb; sequences are described in Table S2 in the supplemental material) were orally delivered for 72 h at 24°C through a Parafilm membrane sachet containing a final concentration of siRNAs of 1 μM in 300 μl of sterilized artificial diet (0.5× AP3 medium [57] supplemented with 20% sucrose). All experimental settings were first determined during *stylin-01* knockdown experiments. To analyze the relative *stylin-01* expression levels, 2 to 4 pools of 10 aphids were collected for each treatment. This experiment was repeated 9 times. In total, 27 pools of 10 aphids were analyzed for NC-siRNA and *sty01*-siRNA treatments. For 2 of these 9 replicates, aphids were additionally collected to perform CaMV transmission tests. To analyze the relative *stylin-02* expression levels, 6 pools of 10 aphids were collected for each treatment, and 3 replicates were performed (in total, 18 pools of 10 aphids were analyzed for each treatment). In all replicates, aphids were additionally collected to perform CaMV transmission tests.

In addition, and to estimate whether silencing of *Stylin-01* reduces P2 binding onto the acrostyle, RNAi treatments were carried out on 3-day-old synchronized A. pisum using the protocol set up for M. persicae (see above). A final concentration of 2 μM siRNA was used, and two independent experiments were performed. Stylets of control and silenced aphids were dissected and incubated with P2-GFP as described above.

### Real-time RT-qPCR.

Total RNA was extracted from pools of 10 aphids using an RNeasy minikit (Qiagen, Hilden Germany). A total of 100 to 400 ng of the total RNA extracted was treated with RQ1 RNase-free DNase I (Promega Corporation, Madison, WI). First-strand cDNA was synthesized using Moloney murine leukemia virus (MMLV) reverse transcriptase (Promega Corporation) according to the manufacturer's instructions, with oligo(dT)_18_ as the primer. Real-time reverse transcription-quantitative PCR (RT-qPCR) was performed on a LightCycler 480 instrument using a LightCycler 480 SYBR green I master mix (Roche, Penzberg, Germany) according to the manufacturer's recommendations with gene-specific primers (listed in Table S2 in the supplemental material). Expression levels were normalized with two internal reference genes encoding actin and elongation factor 1α (EF1α) from M. persicae (58). RT-qPCRs were performed in duplicates. Amplification efficiencies were analyzed with LinRegPCR free software (v. 2014.5) (59). Expression ratios were calculated using the threshold cycle (2^−ΔΔ*CT*^) method (60).

### Transmission tests.

Cohorts of aphids fed for 72 h on artificial diets supplemented with siRNA were collected and starved for 1 h in glass tubes. They were allowed a 5-min acquisition access period (AAP) on CaMV-infected turnips (Brassica rapa cv. Just Right) 23 days postinoculation, followed by a 2-h inoculation access period (IAP) on 8-day-old turnip test plants, before insecticide spray was applied. In Stylin-01 silencing experiments, 2 biological replicates were performed using 2 aphids per test plant and 48 test plants per treatment. In Stylin-02 silencing experiments, 3 biological replicates were performed using 2 aphids per test plant and 72 test plants per treatment. Transmission efficiency was calculated by recording the number of plants presenting symptoms of virus infection 28 days after transmission assay divided by the total number of test plants per treatment. Alternatively, in the case of Stylin-01 silencing experiments, cohorts of aphids fed on artificial diets supplemented with NC-siRNA or *Sty01*-siRNA were collected and attached to a gold wire as previously described (61). Aphids were transferred to CaMV-infected turnip plants after a 1-h starvation period. The aphid feeding behavior was monitored by electrical penetration graphs using an EPG Giga-8 device (EPG Systems, Wageningen, The Netherlands) connected to a USB AD (DI-710; DATAQ Instruments) and a laptop computer. After five intracellular punctures (named as potential drop pattern [pd]), each aphid was transferred to an 8-day-old turnip test plant for a 2-h IAP (1 aphid per plant). Twenty-two aphids were used for each treatment, and the experiment was repeated 5 times. Duration of each pd was analyzed using Stylet+ software (EPG Systems [62]).

### Database searches and sequence retrieval.

For annotating RR-1 cuticular proteins in the genome of Diuraphis noxia (63), we used CutProtFam-Pred (http://aias.biol.uoa.gr/CutProtFam-Pred/ [29]), with the standard settings proposed by the web interface (see Table S3 in the supplemental material). An exhaustive search in Aphididae family was performed with the BLAST programs (64) in the NCBI's (NR, TSA, and SRA) databases and Aphidbase (available at http://www.aphidbase.com/), using A. pisum ACYPI009006 (Stylin-01) and A. pisum ACYPI003649 (Stylin-02) as query sequences. Predicted translation of each full-length or partial retrieved sequence was checked for the presence of the conserved RR-1 motif (GSYSYTxPDGxxYxVxYVAD-ENGFQPxGxHLP) using CuticleDB software (http://biophysics.biol.uoa.gr/cuticleDB/ [65]). All protein sequence analyses (predicted translation and signal peptide searches) were performed using ExPASy tools (http://www.expasy.org/tools/).

### Alignments and phylogenetic reconstruction of the RR-1 family.

Stylin-01 and Stylin-02 homologs were aligned using T-Coffee (66, 67). Phylogenetic relationships between A. pisum, M. persicae, and D. noxia RR-1 proteins were assessed using the Phylogeny.fr platform (68). After removal of the signal peptide, protein sequences of RR-1 genes of A. pisum, M. persicae, and D. noxia were aligned using MUSCLE (v. 3.8.31) (69) configured for highest accuracy (MUSCLE with default settings). Ambiguous regions (i.e., containing gaps and/or poorly aligned) were removed with Gblocks (v. 0.91b) using the following parameters: the minimum length of a block after gap cleaning was 10, no gap positions were allowed in the final alignment, all segments with contiguous nonconserved positions bigger than 8 were rejected, and the minimum number of sequences for a flank position was 85%. Then phylogenetic trees were reconstructed using the maximum likelihood method implemented in the PhyML program (v. 3.1/3.0 aLRT). The WAG substitution model was selected assuming an estimated proportion of invariant sites (of 0.009) and 4 gamma-distributed rate categories to account for rate heterogeneity across sites. The gamma shape parameter was estimated directly from the data (gamma = 3.517). Reliability for internal branch was assessed using the aLRT test (SH-Like). Graphical representation and edition of the phylogenetic tree were performed with TREEDYN (v. 198.3) (70). OrthoMCL software (http://orthomcl.org/orthomcl/) was run on predicted RR-1 protein sets from A. pisum, M. persicae, and D. noxia in order to determine putative orthologs (71).

### Statistical analysis.

All statistical analyses were carried out with R software (v. 3.3.2). Statistical significance for transcript levels and mean duration of potential drop waveforms was assessed by one-way analysis of variance (ANOVA), and CaMV transmissions were compared by performing a chi-square test. Results of each statistical test are indicated in the figure legends.

### Accession number(s).

Stylin-01 sequences from aphid clones maintained in our laboratory have been deposited in GenBank under accession numbers MG188739 to MG188743.

## Supplementary Material

Supplemental material
