# Supplementary material for: Identification of Plant Virus Receptor Candidates in the Stylets of Their Aphid Vectors
Source: J Virol. 2018 Jun 29;92(14):e00432-18. doi: 10.1128/JVI.00432-18 (PMC6026765; doi:10.1128/JVI.00432-18)
Supplement: Supplemental material [file supp_92_14_e00432-18__index.html]

Supplemental material 

# Identification of Plant Virus Receptor Candidates in the Stylets of Their Aphid Vectors

## Supplemental material

- Supplemental file 1 -

  Fig. S1 (Comparison of Stylin-01 and Stylin-02 homologs across aphid species.)

  Table S1 (Accession numbers of sequences used in comparisons presented in Fig. S1.)

  Table S2 (Oligonucleotides used in this study.)

  Table S3 (RR-1 genes of the aphid *Diuraphis noxia* detected using CutProtFam-Pred software.)

  PDF, 4.8M
