# Supplementary material for: Identification of Plant Virus Receptor Candidates in the Stylets of Their Aphid Vectors
Source: J Virol. 2018 Jun 29;92(14):e00432-18. doi: 10.1128/JVI.00432-18 (PMC6026765; doi:10.1128/JVI.00432-18)
Supplement: Supplemental material [file JVI.00432-18_zjv014183697s1.pdf]

Supplemental information

Fig. S1: (Comparison of Stylin-01 and Stylin-02 homologs across aphid species)

Table S1 (Accession numbers of the sequences used in the comparisons presented in Figure S1)

Table S2 (List of oligonucleotides used in this study)

Table S3 (List of RR-1 genes of the aphid *Diuraphis noxia* detected using CutProtFam-Pred software)

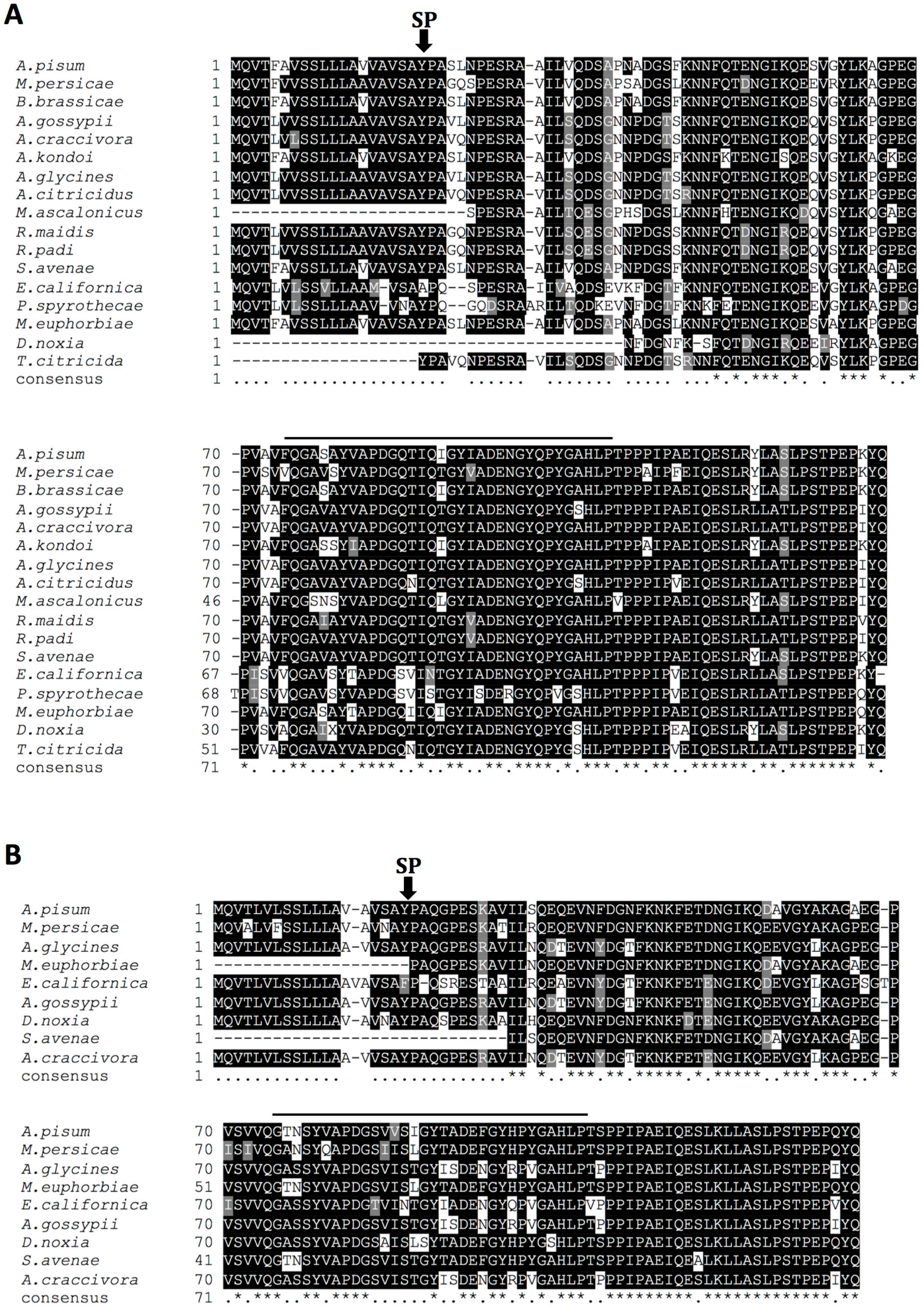

A
SP
A.pisum
M.persicae
B.brassicae
A.gossypii
A.craccivora
A.kondoi
A.glycines
A.citricidus
M.ascalonicus
R.maidis
R.padi
S.avenae
E.californica
P.spyrothecae
M.euphorbiae
D.noxia
T.citricida
consensus
B
SP
A.pisum
M.persicae
A.glycines
M.euphorbiae
E.californica
A.gossypii
D.noxia
S.avenae
A.craccivora
consensus

Figure S1. Comparison of Stylin-01 and Stylin-02 homologs across aphid species.
A comparative analysis was conducted on Stylin-01 and Stylin-02 homologs found in several aphid species (see Table S1 for accession numbers). For this purpose, an exhaustive search in *Aphididae* family was performed with the BLAST programs. In addition, *stylin-01* sequences from aphid clones maintained in our laboratory (*A. pisum LL01*, *Brevicoryne brassicae L., A. gossypii NM1, M. persicae* Sulzer and *A. craccivora*) were obtained from cDNA amplification using corresponding primets sets (See Table S2). Alignments of Stylin-01 (A) and Stylin-02 (B) homologs were performed using the T-Coffee software (1, 2) and shading was done with BOXSHADE 3.21 software. Consensus sequence is shown at the bottom with (.) indicating conserved substitutions and (*) indicating identities. Signal peptide (SP) cleavage site and RR-1 conserved motif (horizontal line) are indicated.

References

1. **Notredame C, Higgins DG, Heringa J.** 2000. T-Coffee: A novel method for fast and accurate multiple sequence alignment. J Mol Biol 302:205-217.
2. **Di Tommaso P, Moretti S, Xenarios I, Orobitg M, Montanyola A, Chang J-M, Taly J-F, Notredame C.** 2011. T-Coffee: a web server for the multiple sequence alignment of protein and RNA sequences using structural information and homology extension. Nucleic Acids Res 39:W13-W17.

Table S1. Accession numbers of the sequences used in the comparisons presented in Figure S1.

Species	Protein	Accession number
*Acyrthosiphon pisum**	Stylin-01	MG188739
*Myzus persicae**	Stylin-01	MG188741
*Brevicoryne brassicae**	Stylin-01	MG188740
*Aphis gossypii**	Stylin-01	MG188742
*Aphis craccivora**	Stylin-01	MG188743
Acyrthosiphon kondoï	Stylin-01	FQ996478
Aphis glycines	Stylin-01	SRX 016521
Aphis citricidus	Stylin-01	CD451041.1 USDA-FP_103086
Myzus ascalonicus	Stylin-01	FO026540
Rhopalosiphum maidis	Stylin-01	FQ977098
Rhopalosiphum padi	Stylin-01	FO061799
Essigella californica	Stylin-01	GAZF02055426
Pemphigus spyrothecae	Stylin-01	FO049664
Macrosiphum euphorbiae	Stylin-01	GAAF01000507
Diuraphis noxia	Stylin-01	XP015364844 (Aphidbase)
Toxoptera citricida	Stylin-01	CTG_TC131_2-17649192 (Aphidbase)
Acyrthosiphon pisum	Stylin-02	ACYPI003649
Myzus persicae	Stylin-02	MYZPE13164_G006_v1.0_000086060 (Aphidbase)
Aphis gossypii	Stylin-02	GW533866.1
Macrosiphum euphorbiae	Stylin-02	SRR547988
Essigella californica	Stylin-02	GAZF02033397
Diuraphis noxia	Stylin-02	XP0153379180 (Aphidbase)
Sitobion avenae	Stylin-02	GAPL01028508
Aphis craccivora	Stylin-02	GAJW01000188

* Sequences obtained in the present study from aphid species maintained in our laboratory

Table S2. List of oligonucleotides used in this study.

Primers' name	Use	sequence (5' to 3')	Comments
pGEX-Ap9006Cter_For<br>pGEX- Ap9006Cter_Rev	Cloning	GATCCccAGATACCTCGCCTCTCTGCCCAGCACCCCCGAACCAAAATACCAGG<br>AATTCCTGGTATTTTGGTTCGGGGGTGCTGGGCAGAGAGGCGAGGTATCTggG	GST-pept 1-11 fusion
pGEX-Ap3649Cter_For<br>pGEX-Ap3649Cter_Rev	Cloning	GATCCccAAATTATTGGCCTCTCTGCCCAGCACACCCGAACCACAGTACCAGG<br>AATTCCTGGTACTGTGGTTCGGGTGTGCTGGGCAGAGAGGCCAATAATTTggG	GST-pept Cter ACYPI003649 fusion
Sty01_For<br>Sty01_Rev	cDNA<br>amplification	AATCACAAACACATCGCACTCCG<br>GTGTGGCRGATRATCAATTCGAGG	*Stylin-01* from *A. pisum,*<br>*M. persicae, B. brassicae*
Ag_Sty01_For<br>Ag_Sty01_Rev	cDNA<br>amplification	CAAACGAACAGAAACTCGTCA<br>TTGCGGATAATGGCTGAAGT	*Stylin-01 from A. gossypii,*<br>*A. craccivora*
qSty01_Mp_For<br>qSty01_Mp_Rev	RT-qPCR<br>RT-qPCR	ACATTTGTCGTATCGTCATTG<br>GCTGGGTGCTGAATCTT	*Stylin-01* from *M. persicae*
qSty02_Mp_For<br>qSty02_Mp_Rev	RT-qPCR<br>RT-qPCR	GGTCGCGTTAGTCTTCTCATCG<br>TGACTTCTTGTTCTTGACGCAG	*Stylin-02* from *M. persicae*
qEF1α _Mp_For<br>qEF1α _Mp_Rev	RT-qPCR<br>RT-qPCR	AAAATGGACAAACCCGTGAA<br>GCTGTATGGTGGTTCAGTAGAA	*EF1-a* from *M. persicae*
qActin_Mp_For<br>qActin_Mp_Rev	RT-qPCR<br>RT-qPCR	CGTTACCAACTGGGACGATATG<br>GGGTTCAATGGAGCTTCTGTAA	*Actin* from *M. persicae*
NC-siRNA	Silencing	Reference SR-CL00-005 (Kaneka Eurogentec S.A., Seraing, Belgium)	siRNA duplex negative control
sty01-siRNA sense	Silencing	AUCCCAUUCGAGAUCCAAG (+dTdT)	Targeting *stylin-01* mRNA from<br>*M. persicae.*
sty02-siRNAa sense<br>*sty02*-siRNAb sense	Silencing<br>Silencing	UCCUGCGUCAAGAACAAGA (+dTdT)<br>UUGUCCAGGGAGCAAAUUC (+dTdT)	Targeting *stylin-02* mRNA from<br>*M. persicae.*

5' and 3' overhanging sequences of *Bam*HI and *Eco*RI restrictions sites are underlined. Bases added to keep the open reading frame are in lower cases.

Table S3. List of RR-1 genes of the aphid *Diuraphis noxia* detected using CutProtFam-Pred software.

Sequence Name	Family Type	E-value	Score	Previous annotation description*
XP_015364772.1	CPR_RR-1	7.10E-12	38	PREDICTED: foot protein 1 variant 1
XP_015364844.1	CPR_RR-1	3.00E-18	59.2	PREDICTED: endocuticle structural glycoprotein SgAbd-4-like, partial
XP_015365268.1	CPR_RR-1	4.80E-09	28.4	PREDICTED: flexible cuticle protein 12-like
XP_015371382.1	CPR_RR-1	3.70E-08	20	PREDICTED: uncharacterized protein LOC107167013
XP_015372692.1	CPR_RR-1	5.70E-08	18.3	PREDICTED: uncharacterized protein LOC107167970
XP_015375796.1	CPR_RR-1	4.70E-14	45.3	PREDICTED: uncharacterized protein LOC107170242
XP_015378979.1	CPR_RR-1	1.50E-16	53.6	PREDICTED: LOW QUALITY PROTEIN: larval cuticle protein LCP-30-like
XP_015379180.1	CPR_RR-1	2.20E-21	69.6	PREDICTED: endocuticle structural glycoprotein SgAbd-4-like
XP_015379719.1	CPR_RR-1	2.00E-20	66.4	PREDICTED: paternally-expressed gene 3 protein-like
XP_015379744.1	CPR_RR-1	3.60E-19	62.3	PREDICTED: endocuticle structural glycoprotein ABD-4-like
XP_015379794.1	CPR_RR-1	2.10E-19	63	PREDICTED: endocuticle structural glycoprotein SgAbd-8-like
XP_015379869.1	CPR_RR-1	6.40E-22	71.4	PREDICTED: probable serine/threonine-protein kinase fhkB

* Predicted by NCBI's automated computational analysis derived from genomic sequences (1) annotated using gene prediction method: Gnomon, supported by EST evidence

Reference

1. **Nicholson SJ, Nickerson ML, Dean M, Song Y, Hoyt PR, Rhee H, Kim C, Puterka GJ.** 2015. The genome of Diuraphis noxia, a global aphid pest of small grains. BMC Genomics, 16: 429 doi: 10.1186/s12864-015-1525-1
